# Association of Cigarette Smoking With Cerebrospinal Fluid Biomarkers of Neurodegeneration, Neuroinflammation, and Oxidation

**DOI:** 10.1001/jamanetworkopen.2020.18777

**Published:** 2020-10-02

**Authors:** Yanlong Liu, Hui Li, Jian Wang, Qing Xue, Xiaoyu Yang, Yimin Kang, Mengjie Li, Jinzhong Xu, Guohua Li, Cunbao Li, Hui-Chih Chang, Kuan-Pin Su, Fan Wang

**Affiliations:** 1 School of Mental Health, Wenzhou Medical University, Wenzhou, China; 2 The Affiliated Kangning Hospital, Wenzhou Medical University, Wenzhou, China; 3 College of Pharmaceutical Sciences, Wenzhou Medical University, Wenzhou, China; 4 Department of Biomedical Engineering, College of Engineering, Peking University, Beijing, China; 5 Xinjiang Key Laboratory of Neurological Disorder Research, Second Affiliated Hospital of Xinjiang Medical University, Urumqi, China; 6 Psychosomatic Medicine Research Division, Inner Mongolia Medical University, Huhhot, China; 7 Department of Psychology, Guang’anmen Hospital, China Academy of Chinese Medical Sciences, Beijing, China; 8 Fifth Affiliated Hospital of Sun Yat-sen University, Zhuhai, China; 9 Beijing Jishuitan Hospital, Beijing, China; 10 Sleep Medicine Center, Peking University International Hospital, Beijing, China; 11 Affiliated Wenling Hospital of Wenzhou Medical University, Wenling, China; 12 School of Public Health and Management, Wenzhou Medical University, Wenzhou, China; 13 Department of Psychiatry and Mind-Body Interface Laboratory, China Medical University Hospital, Taichung, Taiwan; 14 College of Medicine, China Medical University, Taichung, Taiwan; 15 An-Nan Hospital, China Medical University, Tainan, Taiwan; 16 Beijing Hui-Long-Guan Hospital, Peking University, Beijing, China

## Abstract

**Importance:**

Cigarette smoking has been associated with risk of neurodegenerative disorders, such as Alzheimer disease. The association between smoking and biomarkers of changes in human cerebrospinal fluid (CSF) is not fully understood.

**Objective:**

To investigate the association of cigarette smoking with CSF biomarkers of neurodegeneration, neuroinflammation, oxidation, and neuroprotection.

**Design, Setting, and Participants:**

In this case-control study of 191 adult men in China, biomarkers in the CSF of participants with and without significant cigarette exposure were examined. Participants who did not smoke and had no history of substance use disorder or dependence were assigned to the nonsmoking group. The active smoking group included participants who consumed at least 10 cigarettes per day for 1 year. Five-milliliter samples of CSF were obtained from routine lumbar puncture conducted before anterior cruciate ligament reconstruction surgery. Data collection took place from September 2014 to January 2016, and analysis took place from January to February 2016.

**Exposures:**

Cigarette smoking.

**Main Outcomes and Measures:**

CSF levels of β-amyloid 42 (Aβ42), which has diagnostic specificity for Alzheimer disease, tumor necrosis factor alpha (TNFα), brain-derived neurotrophic factor (BDNF), total superoxide dismutase (SOD), and nitric oxide synthase (NOS) were measured. Sociodemographic data and history of smoking were obtained.

**Results:**

Of 191 participants, 87 (45.5%) were included in the active smoking group and 104 (54.4%) in the nonsmoking group. Compared with the active smoking group, the nonsmoking group was younger (mean [SD] age, 34.4 [10.5] years vs 29.6 [9.5] years; *P* = .01), had more education (mean [SD] duration of education, 11.9 [3.1] years vs 13.2 [2.6] years; *P* = .001), and had lower body mass index (mean [SD], 25.9 [3.6] vs 24.9 [4.0]; *P* = .005). Comparing the nonsmoking group with the smoking group, mean (SD) CSF levels of Aβ42 (38.0 [25.9] pg/mL vs 52.8 [16.5] pg/mL; *P* < .001) and TNFα (23.0 [2.5] pg/mL vs 28.0 [2.0] pg/mL; *P* < .001) were significantly lower, while BDNF (23.1 [3.9] pg/mL vs 13.8 [2.7] pg/mL; *P* < .001), total SOD (15.7 [2.6] U/L vs 13.9 [2.4] U/L; *P* < .001), total NOS (28.3 [7.2] U/L vs 14.7 [5.6] U/L; *P* < .001), inducible NOS (16.0 [5.4] U/L vs 10.3 [2.7] U/L; *P* < .001), and constitutive NOS (12.4 [6.9] U/mL vs 4.4 [3.9] U/mL) were higher. In addition, in participants in the smoking group who were aged 40 years or older, total SOD levels were negatively correlated with Aβ42 levels (*r* = −0.57; *P* = .02). In those who smoked at least 20 cigarettes per day, TNFα levels were positively correlated with Aβ42 levels (*r* = 0.51; *P* = .006). The association of TNFα with Aβ42 production was stronger than that of total SOD with Aβ42 production (*z* = −4.38; *P* < .001).

**Conclusions and Relevance:**

This case-control study found that cigarette smoking was associated with at-risk biomarkers for Alzheimer disease, as indicated by higher Aβ42 levels, excessive oxidative stress, neuroinflammation, and impaired neuroprotection found in the CSF of participants in the active smoking group.

## Key Points

**Question:**

Is cigarette smoking associated with changes in specific neurodegenerative biomarkers in cerebrospinal fluid?

**Findings:**

In this case-control study of 191 adult men in China, active smoking was associated with at-risk biomarkers for Alzheimer disease, as indicated by higher β-amyloid 42 levels.

**Meaning:**

The results of this study broaden our understanding of the association of cigarette smoking with neurodegenerative disorders.

## Teaser

This case-control study investigates the association of cigarette smoking with cerebrospinal fluid biomarkers of neurodegeneration, neuroinflammation, oxidation, and neuroprotection.

## Introduction

Evidence from epidemiological studies and meta-analyses have indicated that cigarette smoking is significantly associated with the risk of neurodegenerative disorders,^1,2,3^ including Alzheimer disease (AD) and dementia. It has been reported that heavy smoking is associated with a greater than 100% increase in risk of dementia and AD after 2 decades of exposure.^4^ With memory loss and cognitive deficit, AD is among the most devastating brain disorders for older individuals.^5^ It will affect approximately 100 million people worldwide by 2050.^6^ Nevertheless, the risk factors and the pathophysiology of AD have not been fully understood.

Cigarette smoking exacerbates amyloid pathology in an animal model of AD.^7^ The measurement of β-amyloid 42 (Aβ42) levels in cerebrospinal fluid (CSF) has diagnostic specificity for AD.^8,9,10^ Human studies have demonstrated that high CSF Aβ42 levels are strongly associated with AD and mild cognitive impairment due to AD.^11^ The primary hypothesis for AD development suggests that Aβ42 promotes plaque formation, accompanied with oxidative stress, cortical inflammation, and neuronal loss,^12,13^ while the abnormal deposition of protein aggregation causes neurotoxicity, cell death, and neurodegeneration.^14,15^ Furthermore, individuals with mild cognitive impairment showed increased brain oxidative damage before the onset of symptomatic dementia.^16^ It is understood that the combined effects of oxidative stress and neuroinflammation lead to the accumulation of Aβ,^17^ and antioxidant supplement application might slow down functional decline in patients with AD.^18^ Animal models suggest a direct and causal relationship between increased Aβ, reduced superoxide dismutase (SOD) activity, and oxidative damage in AD.^19^ The reaction of nitric oxide and superoxide anion might be responsible for the cellular damage in neurodegenerative disorders, which is a widely accepted explanation of AD.^20,21^ Nitric oxide is associated with cognitive function,^20^ and it plays an obligatory role in neuroprotection in the central nervous system (CNS).^22^ Nitric oxide synthase (NOS) catalyzes nitric oxide formation and accelerates reactions that reduce oxidation. Purified NOS directly catalyzes the generation of oxygen radicals.^23^ NOS is upregulated in patients with AD, suggesting that these enzymes are instrumental in the pathogenesis of this disease.^24^ Cigarette smoking has been associated with increased production of reactive oxygen species,^25,26^ stimulating pro-inflammatory gene transcription and the release of cytokines, such as tumor necrosis factor α (TNFα), which is associated with further increases in Aβ formation.^27,28^

Increased levels of reactive oxygen species and neuroinflammation are associated with decreases in brain-derived neurotrophic factor (BDNF) expression.^29,30,31,32^ Previous studies have suggested that lower concentrations of CSF BDNF are associated with the progression from mild cognitive impairment to AD.^33^ The neurotrophin BDNF has been shown to protect against future occurrence of dementia and AD and to decrease the risk of dementia in some human studies.^34,35,36^ Nevertheless, cigarette smoking showed different associations with BDNF levels in human and animal studies.^37,38^ The present study was conducted to investigate the CSF levels of Aβ42, oxidative stress, neuroinflammation, and neuroprotection in individuals who actively smoke and to further explore the association of cigarette smoking with AD.

## Methods

### Study Design

This case-control study was conducted following the Strengthening the Reporting of Observational Studies in Epidemiology (STROBE) reporting guideline. This study was approved by the institutional review board of Inner Mongolian Medical University and was performed in accordance with the Declaration of Helsinki.^39^ Written informed consent was obtained from all participants, and no financial compensation was provided to the participants. The cases were defined as individuals who actively smoke, and controls were matched individuals who do not smoke.

### Participants

Cigarette smoking is common; however, the association of cigarette smoking with AD is unclear, and it is difficult to collect CSF. Therefore, a sample size of more than 70 participants per group represents a relatively large sample.

Because there are few women who smoke in China,^40^ very few women were recruited to the active smoking group. Therefore, a total of 191 men in China who were scheduled for anterior cruciate ligament reconstruction surgery were recruited from September 2014 to January 2016. Overall, 87 actively smoked, while 104 did not. The active smoking group was further divided into a young subgroup (ie, aged <40 years) and a midlife subgroup (ie, aged ≥40 years), according to the standard diagnostic manual of the American Psychiatric Association. In addition, those with moderate tobacco use (ie, <20 cigarettes/day) were compared with those with heavy tobacco use (≥20 cigarettes/day), according to World Health Organization criteria.

Sociodemographic data, including age and years of education, were collected. Clinical data, such as history of substance use disorders and dependence, were obtained according to self-report and confirmed by next of kin and family members. Patients with a family history of psychosis and neurological diseases or systemic or CNS diseases, determined by the Mini-International Neuropsychiatric Interview, were excluded.

Participants without a history of any substance use disorder or dependence, including cigarettes, were assigned to nonsmoking group. The active smoking group included participants who consumed at least 10 cigarettes per day for 1 year. Few participants had history of alcohol use disorder, and all participants had no other psychiatric disorders according to the *Diagnostic and Statistical Manual of Mental Disorders* (Fourth Edition).

### Assessments, Biological Sample Collection, and Laboratory Tests

Smoking-related information was obtained from those who actively smoked, including age at smoking onset, years of active smoking, mean number of cigarettes smoked per day, and maximum number of cigarettes smoked per day. Lumbar puncture is part of standard clinical practice for patients undergoing anterior cruciate ligament reconstructive surgery. Lumbar puncture was performed in the morning before surgery by a licensed anesthetist, and a 5-mL CSF sample was obtained via intrathecal collection. The time between assessment and lumbar puncture was less than 24 hours. Each CSF sample was then distributed in ten 0.5-mL tubes and immediately frozen at –80 °C for storage.

CSF levels of Aβ42 and BDNF were measured using enzyme-linked immunosorbent assay kits (Phoenix Pharmaceuticals and Cloud-clone, respectively); TNFα, radioimmunoassay kits (DIAsource ImmunoAssays); and total SOD and NOS, spectrophotometric kits (Nanjing Jiancheng Bioengineering Institute). Laboratory technicians were masked to clinical data.

### Statistical Analysis

General demographic and clinical data as well as raw biomarker data were compared between groups using the Wilcoxon rank sum test. Analysis of covariance was used for continuous variables to assess the differences between groups. Pearson correlation and partial correlation analysis were performed to test continuous variables and smoking status, adjusted for the covariate of years of education.

We used analysis of covariance to estimate the association of cigarette smoking with each variable, adjusting for age, years of education, and other biomarkers. To explore the association of SOD and TNFα levels with Aβ42 production, partial correlation was performed, adjusted for years of education. To calculate the difference between the 2 *r* values, Fisher *r*-to-*z* exchange was used, based on the literature.^41^ All statistical analyses were performed using SPSS statistical software version 20.0 (IBM Corp). Figures were created using Prism version 6 (GraphPad) and R version 3.6.1 (R Project for Statistical Computing). All tests were 2-sided, and the significance threshold was set at *P* < .05.

## Results

### Demographic and Clinical Characteristics

Of 191 participants, 87 (45.5%) were included in the active smoking group and 104 (54.4%) in the nonsmoking group. Participants in the nonsmoking group were younger (mean [SD] age, 34.4 [10.5] years vs 29.6 [9.5] years; *P* = .01), had more education (mean [SD] duration of education, 11.9 [3.1] years vs 13.2 [2.6] years; *P* = .001), and had lower mean (SD) body mass index, calculated as weight in kilograms divided by height in meters squared (25.9 [3.6] vs 24.9 [4.0]; *P* = .005), while no differences were found in terms of clinical characteristics between the 2 groups (Table 1).

**Table 1.** Clinical Characteristics of Groups

Characteristic	Mean (SD)		*P* value
	Nonsmoking group (n = 104)	Active smoking group (n = 87)	
Age, y	29.6 (9.5)	34.4 (10.5)	.01
Education, y	13.2 (2.6)	11.9 (3.1)	.001
BMI	24.9 (4.0)	25.9 (3.6)	.005
Systolic blood pressure, mm Hg	129.8 (12.8)	127.6 (13.5)	.25
Diastolic blood pressure, mm Hg	75.2 (9.4)	76.9 (11.6)	.30
HDL, mg/dL	50.2 (11.6)	46.3 (11.6)	.36
LDL, mg/dL	104.2 (27.0)	104.2 (23.3)	.89
ALT, U/L	30.0 (22.8)	31.5 (22.9)	.70
Cholesterol, mg/dL	181.5 (38.6)	185.3 (30.9)	.44
Triglycerides, mg/dL	159.3 (97.3)	159.3 (115.0)	.28
GGT, U/L	40.4 (30.8)	47.3 (45.6)	.43
AST, U/L	21.5 (9. 4)	20.6 (7.4)	.66
CSF Aβ42, pg/mL	38.0 (25.9)	52.8(16.5)	<.001
CSF BDNF, pg/mL	23.1 (3.9)	13.8 (2.7)	<.001
CSF total SOD, U/mL	15.7 (2.6)	13.9 (2.4)	<.001
CSF total NOS, U/mL	28.3 (7.2)	14.7 (5.6)	<.001
CSF inducible NOS, U/mL	16.0 (5.4)	10.3 (2.7)	<.001
CSF constitutive NOS, U/mL	12.4 (6.9)	4.4 (3.9)	<.001
CSF TNFα, pg/mL	23.0 (2.5)	28.0 (2.0)	<.001

Abbreviations: Aβ42, amyloid beta 42; ALT, alanine aminotransferase; AST, aspartate aminotransferase; BDNF, brain-derived neurotrophic factor; BMI, body mass index (calculated as weight in kilograms divided by height in meters squared); CSF, cerebral spinal fluid; GGT, γ-glutamyltransferase; HDL, high-density lipoprotein; LDL, low-density lipoprotein; NOS, nitric oxide synthase; SOD, superoxide dismutase; TNFα, tumor necrosis factor α.

SI conversion factors: To convert ALT, AST, and GGT to microkatals per liter, multiply by 0.0167; BDNF to nanograms per liter, multiply by 1.0; cholesterol, HDL, and LDL to micromoles per liter, multiply by 0.0259; and triglycerides to micromoles per liter, multiply by 0.0113.

### CSF Biomarkers

**Figure 1.**
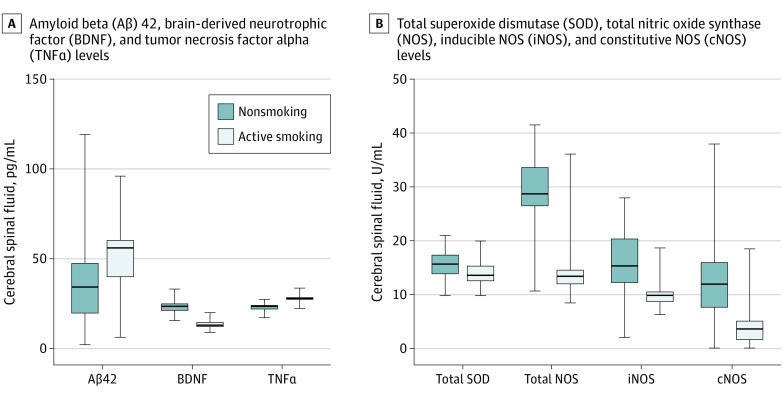
Differences in Biomarker Levels in Cerebral Spinal Fluid Between Active Smoking and Nonsmoking Groups To convert BDNF to nanograms per liter, multiply by 1.0.

Using analysis of covariance with age, years of education, and other biomarkers as covariates and comparing the nonsmoking group with the active smoking group, mean (SD) CSF levels of TNFα (23.0 [2.5] pg/mL vs 28.0 [2.0] pg/mL; *P* < .001) and Aβ42 (38.0 [25.9] pg/mL vs 52.8 [16.5] pg/mL; *P* < .001) were significantly lower in the nonsmoking group. However, BDNF (23.1 [3.9] pg/mL vs 13.8 [2.7] pg/mL; *P* < .001 [to convert to nanograms per liter, multiply by 1.0]), total SOD (15.7 [2.6] U/L vs 13.9 [2.4] U/L; *P* < .001), total NOS (28.3 [7.2] U/L vs 14.7 [5.6] U/L; *P* < .001), inducible NOS (16.0 [5.4] U/L vs 10.3 [2.7] U/L; *P* < .001), and constitutive NOS levels (12.4 [6.9 U/mL vs 4.4 [3.9] U/mL) were higher in the nonsmoking group (Table 2 and Figure 1).

**Table 2.** Differences in Cerebrospinal Fluid Biomarker Levels Between Nonsmoking and Active Smoking Group

Category	Mean difference (95% CI)	*P* value
Dementia related		
Aβ42, pg/mL	–18.5 (–34.4 to –2.6)	.02
Neurotrophin		
BDNF, pg/mL	9.6 (7.6 to 11.5)	<.001
Oxidation		
Total SOD, U/mL	2.1 (0.3 to 3.8)	.02
Total NOS, U/mL	13.1 (8.6 to 17.5)	<.001
Inducible NOS, U/mL	3.7 (0.7 to 6.8)	.02
Constitutive NOS, U/mL	11.2 (7.3 to 15.1)	<.001
Neuroinflammation		
TNFα, pg/mL	–4.7 (–6.2 to –3.2)	<.001

Abbreviations: Aβ42, β-amyloid 42; BDNF, brain-derived neurotrophic factor; NOS, nitric oxide synthase; SOD, superoxide dismutase; TNFα, tumor necrosis factor α.

SI conversion factor: To convert BDNF to nanograms per liter, multiply by 1.0.

### Difference and Correlation in Active Smoking Group

The 87 participants in the active smoking group were divided into young (62 [71.3%]) and midlife (25 [28.7%]) subgroups; 51 (58.6%) were classified as having moderate smoking and 36 (41.4%) as having heavy smoking. There were no differences in CSF levels of BDNF, total SOD, total NOS, inducible NOS, constitutive NOS, and Aβ42 between the young and midlife groups nor between those with moderate and heavy smoking, after adjusting for age, years of education, other biomarkers, and smoking habit (Table 3).

**Table 3.** Differences in Cerebrospinal Fluid Biomarker Levels Among Subgroups of Active Smoking Group

Biomarker	Young vs midlife participants^a^		Moderate vs heavy smoking^b^	
	Mean difference (95% CI)	*P* value	Mean difference (95% CI)	*P* value
Aβ42, pg/mL	6.8 (–11.6 to 24.1)	.49	–1.1 (–15.3 to 13.0)	.87
BDNF, pg/mL	–0.1 (–2.9 to 2.6)	.93	0.2 (–1.9 to 2.3)	.87
Total SOD, U/mL	–1.0 (–3.5 to 1.5)	.41	0.7 (–1.3 to 2.7)	.50
Total NOS, U/mL	2.0 (–4.9 to 8.9)	.57	–0.2 (–5.6 to 5.2)	.94
Inducible NOS, U/mL	0.9 (–1.7 to 3.6)	.49	–1.2 (–3.3 to 0.9)	.25
Constitutive NOS, U/mL	–0.2 (–4.2 to 3.9)	.93	1.5 (–1.6 to 4.6)	.34
TNFα, pg/mL	0.6 (–1.5 to 2.7)	.58	0.5 (–1.1 to 2.2)	.52

Abbreviations: Aβ42, β-amyloid 42; BDNF, brain-derived neurotrophic factor; NOS, nitric oxide synthase; SOD, superoxide dismutase; TNFα, tumor necrosis factor α.

SI conversion factor: To convert BDNF to nanograms per liter, multiply by 1.0.

^a^ Overall, 62 (71.3%) participants were younger than 40 years and included in the young subgroup, and 25 (28.7%) were included in the midlife subgroup.

^b^ Overall, 51 (58.6%) participants smoked fewer than 20 cigarettes a day and were included in the moderate smoking subgroup, and 36 (41.4%) participants were included in the heavy smoking subgroup.

**Figure 2.**
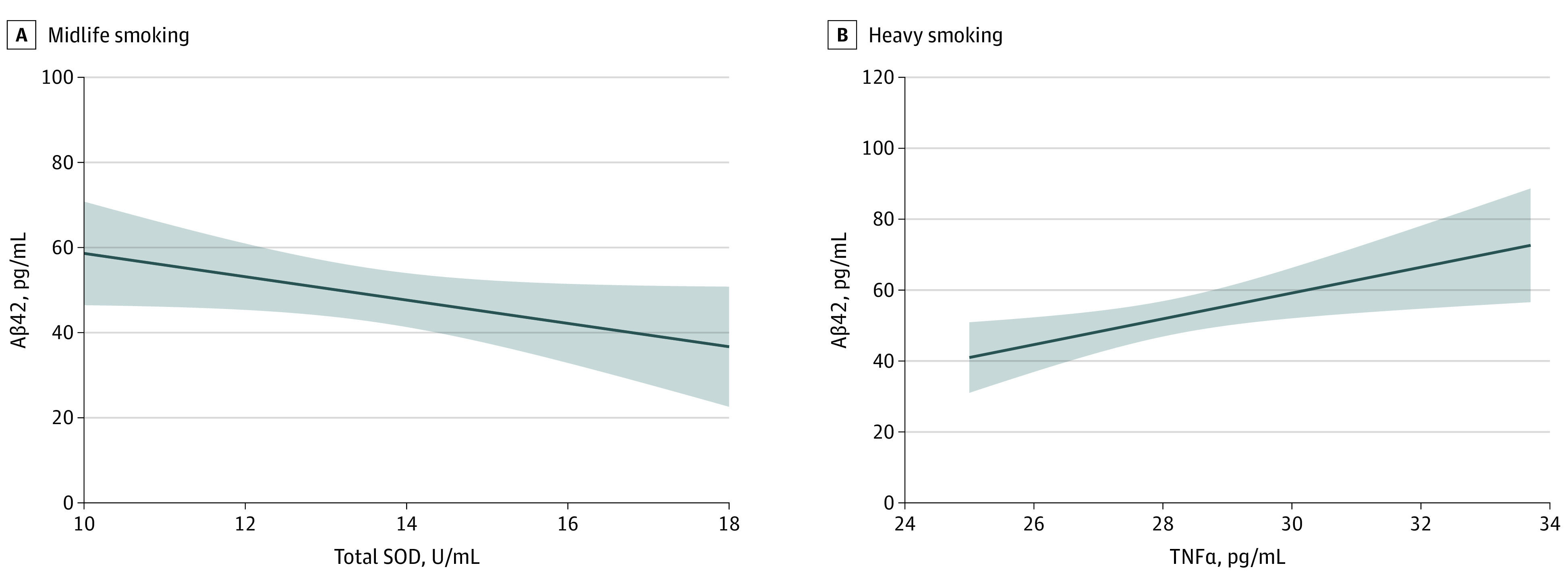
Correlation of β-Amyloid 42 (Aβ42) Levels With Total Superoxide Dismutase (SOD) and Tumor Necrosis Factor α (TNFα) Levels A, The negative correlation of Aβ42 levels with total SOD levels in participants aged 40 years or older who actively smoke (*r* = −0.57; *P* = .02). B, The positive correlation of Aβ42 levels with TNFα levels in those who smoke at least 20 cigarettes per day (*r* = 0.51; *P* = .006).

Total SOD levels were negatively correlated with Aβ42 levels (*r* = −0.57; *P* = .02) in the midlife group (Figure 2A), and TNFα levels were positively correlated with Aβ42 levels (*r* = 0.51; *P* = .006) in those with heavy smoking (Figure 2B), suggesting that the association of TNFα with Aβ42 production was stronger than that of total SOD (*z* = −4.38; *P* < .001). There were no significant correlations between AD CSF biomarkers and age at smoking onset, years of cigarettes smoked, mean number of cigarettes smoked per day, and maximum number of cigarettes smoked per day, adjusted for years of education.

## Discussion

To our knowledge, this is the first case-control study to investigate the association of cigarette smoking with biomarkers of neurodegeneration, oxidation, and neuroinflammation using CSF. The primary finding was that cigarette smoking was associated with at-risk biomarkers for AD, as shown by higher Aβ42 levels from CSF of participants in the active smoking group. This finding further supports the notion that cigarette smoking is among the risk factors for AD. Rapid changes of CSF Aβ42 levels in older adults with normal cognitive function has indicated the emergence of Aβ42 pathology.^42,43^ CSF levels of Aβ42 have been shown to have diagnostic accuracy for AD,^44^ given that changes in these levels are observed years before cognitive decline,^45,46,47^ allowing for the identification of preclinical AD.^48^ AD first causes Aβ deposition in the neocortex and later causes tangles in the temporal lobe, sometimes in addition to age-related tangles.^49^ A large cohort study^4^ has shown that the risk of dementia and AD is dose-dependent, in that risk increases with the increasing number of cigarettes smoked. Indeed, those with very heavy smoking habits are at the greatest risk of dementia, even decades later in life. Moreover, smoking is a significant risk factor for AD,^50^ and cigarette smoking increases the severity of some typical abnormalities of AD, including amyloid genesis.^7^

The secondary findings demonstrated higher levels of total SOD, total NOS, constitutive NOS, and inducible NOS as well as lower levels of TNFα in CSF of participants in the nonsmoking group, indicating neuroinflammation with excessive oxidative stress in participants in the active smoking group. Smoking-related cerebral oxidative stress might serve as a fundamental mechanism contributing to neurobiological abnormalities.^51,52,53^

Previous research has revealed that the accumulation of some inflammatory toxins from cigarette smoke induces lung injury,^54^ eg, lipopolysaccharide,^55^ which not only disrupts the blood-brain barrier^56^ but also activates microglia.^57^ The activated classical microglia can further produce pro-inflammatory cytokines and inflammatory mediators, which elicit toxic effects on neurons and promote tissue inflammation and damage.^58^ The gas and particulate phases of cigarette smoking have extremely high concentrations of short-lived and long-lived reactive oxygen species and other oxidizing agents, which have been believed to be associated with the etiology of AD.^17^ In addition to increased free radical concentrations, cigarette smoking is associated with markedly altered mitochondrial respiratory chain function and the induction of pro-inflammatory cytokines, released by peripheral and CNS glial cells, which collectively promote significant cerebral oxidative stress.^1^ Cigarette smoking also affects the immune system and augments the production of TNFα,^59^ which can increase Aβ burden through the upregulation of β-secretase production.^27,28^ Overproduction of nitrous oxide in the CNS is thought to play a critical role in the pathology of AD.^60^ Constitutive NOS consists of endothelial NOS and neuronal NOS. Endothelial NOS activity is inhibited by Aβ in basal phosphorylation,^61^ and neuronal NOS is inhibited by cigarette smoking.^62^ During CNS inflammation, the increase in endogenous inflammatory cytokine concentration can induce inducible NOS,^63^ and a rat model of systemic inflammation^64^ indicated that increased systemic inducible NOS activity can reverse cognitive deficits. Previous studies have reported that chemicals within cigarette smoke reduce the expression of glial inducible NOS.^65^ In the present study, we found higher TNFα levels but lower total SOD, total NOS, constitutive NOS, and inducible NOS in the CSF of participants in the active smoking group, which indicates that cigarette smoking increases the risk of AD in different ways.

Other findings of the current study included that total SOD levels were negatively correlated with Aβ42 levels in those who were aged 40 years or older and actively smoked. TNFα levels were positively correlated with Aβ42 levels in those who smoked at least 20 cigarettes a day. Aβ impairs mitochondrial function and energy homeostasis in vivo and may directly interact with mitochondria.^66^ Normally, Aβ decreases as antioxidant concentrations increase.^67^ In those who smoke, SOD as an antioxidant in vivo is consumed ceaselessly because of overproduction of oxidants. Previous studies have consistently demonstrated age-related increases of oxidative stress^68,69^ and reported that heavy smoking in midlife was associated with a 100% increase in the risk of dementia and AD.^4^ These findings strongly support our finding that total SOD levels were negatively correlated with Aβ42 levels in those who smoked and were aged 40 years or older. TNFα has been shown to increase the Aβ burden through suppression of Aβ clearance,^28^ which provides an explanation for why TNFα levels were positively correlated with the Aβ42 levels in those with heavy smoking. Importantly, using 2 *r* value comparisons, we found that the association of TNFα with Aβ42 production was stronger than that of total SOD. This comparison provided indirect evidence that pro-inflammatory TNFα exerted more contribution to the at-risk AD pathogenesis than SOD.

### Limitations

There were some limitations for the present study. First, CSF cannot directly reflect neuron changes in AD pathology; nevertheless, it represents biochemical changes in the brain. Second, CSF biomarkers measured in this study are considered risk factors for AD instead of biomarkers related to AD pathology with up-to-date knowledge. Third, participants recruited in this study were patients with anterior cruciate ligament injuries instead of healthy individuals, which might be seen as a confounder when interpreting the results.

## Conclusions

In this study, cigarette smoking was associated with CSF biomarkers of neurodegeneration, neuroinflammation, and oxidation. These are associated with risk of AD.
